# The rise of marine fungi: Drug discovery's blue frontier

**DOI:** 10.1002/ctm2.70623

**Published:** 2026-04-12

**Authors:** Eva Breyer, Federico Baltar

**Affiliations:** ^1^ Fungal and Biogeochemical Oceanography Group, College of Oceanography and Ecological Sciences Shanghai Ocean University Nanhui New City China

1

The global burden of antimicrobial resistance underscores a critical stagnation in our ability to discover novel antibiotics.^1^, ^2^ While natural products and their derivatives have historically been the most prolific source of new drugs, the search for novel chemical scaffolds demands new ecological frontiers.^3^ Our recent quantification of pelagic fungi as a major component of ocean biomass reveals just such a frontier—an immense, genetically diverse, and virtually untapped resource poised to reinvigorate the translational pipeline from marine ecology to clinical practice.^4^ This potential is not merely theoretical; it is exemplified by marine fungal‐derived metabolites like Plinabulin, which has advanced through clinical trials,^5^ and is grounded in genomic data revealing a rich, uncharted landscape of biosynthetic potential, for example, vitamin production, within these oceanic communities.^6^

Harnessing this potential first requires overcoming the well‐known ‘great plate count anomaly,’ which has left the majority of marine microbial diversity uncultured and its chemical repertoire unknown.^7^ Yet, our findings transform pelagic fungi from a methodological challenge into a quantifiable opportunity.^4^ Their established biomass proves they are not a rare novelty but a ubiquitous and major component of marine ecosystems, representing a systematic, rather than sporadic, new front in the search for bioactive compounds. This systematic significance is further highlighted by marine natural product research, which has a proven track record of delivering unique scaffolds. Notably, from 2019 to 2023, fungi were the source of the highest number of newly discovered compounds among all marine organisms, underscoring the ocean's potential as a discovery engine and the particular promise of its fungal inhabitants.^8^

The timing for a focused exploration of marine fungi is uniquely favourable, converging with a paradigm shift in discovery science. The traditional one‐molecule‐at‐a‐time approach is being superseded by holistic strategies that integrate multi‐omics data with high‐throughput experimentation. For pelagic fungi, this means their environmental genomes (metagenomes and metatranscriptomes) are no longer just ecological records but become directly searchable blueprints for biosynthesis. This allows researchers to prioritise target organisms not by chance, but by genetic signature, filtering for those with a high density of biosynthetic gene clusters (BGCs) adjacent to stress‐response elements or horizontal gene transfer markers—genomic hallmarks of adaptive, potent chemistry evolved in competitive marine niches.^9^

Fortunately, modern translational science provides the tools to bridge this gap. A targeted pipeline can now be envisioned: first, genome‐mining of eukaryotic metagenomes guides the targeted cultivation of fungi harbouring prolific BGCs.^9^ Subsequently, culture‐based elicitation strategies, such as co‐cultivation with bacterial competitors, can mimic ecological interactions to unlock the production of cryptic antimicrobial compounds.^10^ Finally, artificial intelligence‐driven screening of the resulting chemical libraries against resistant pathogens or specific cancer cell lines offers an unprecedented method to prioritise leads with clinical relevance.^11^

This pipeline culminates in a tangible workflow for collaboration. Imagine a scenario initiated by marine biologists isolating a deep‐sea fungus, predicted via genome mining to possess novel polyketide synthase BGCs. This strain is then co‐cultured with antibiotic‐resistant *Pseudomonas aeruginosa* isolates provided by clinical microbiologists, triggering the production of defensive metabolites. The extracted compounds are screened not just for general toxicity, but against a panel of genetically defined, multidrug‐resistant ESKAPE pathogens curated by infectious disease specialists. Hits are further prioritised by artificial intelligence (AI) models trained on molecular structures and known mechanisms of action. This closed‐loop, from ecological discovery to clinically relevant assay, exemplifies the integrated model needed to accelerate translation.

The path forward is clear. We now recognise pelagic fungi not merely as ecological curiosities, but as a vast, quantifiable reservoir of chemical innovation. Combined with the pressing need underscored by antimicrobial resistance and the maturation of targeted discovery platforms, this presents a unique translational opportunity. To convert this potential into clinical reality, we advocate for structured collaborations where marine microbiologists, natural product chemists, and clinical researchers co‐design workflows from inception. By integrating ecological insight with genomic prediction, innovative cultivation, and AI‐powered screening against specific medical targets, we can systematically explore this new frontier. The ocean's fungal biomass represents one of our most promising untapped sources for the next generation of antimicrobials and anticancer agents—it is time for the clinical community to dive in.

## AUTHOR CONTRIBUTIONS

Eva Breyer wrote the original draft. Eva Breyer and Federico Baltar contributed to the review & editing of the final manuscript.

## CONFLICT OF INTEREST STATEMENT

The authors declare no conflict of interest.
